# Effects of increasing compliance with minimal sedation on duration of mechanical ventilation: a quality improvement intervention

**DOI:** 10.1186/cc11335

**Published:** 2012-05-08

**Authors:** Andre CKB Amaral, Lars Kure, Angie Jeffs

**Affiliations:** 1 Department of Critical Care Medicine, Sunnybrook Health Sciences Centre, 2075 Bayview Ave, Toronto, M4N 3M5, Canada; 2 Department of Medicine, Interdepartmental Division of Critical Care Medicine, University of Toronto, 2075 Bayview Ave, Toronto, M4N 3M5, Canada

## Abstract

**Introduction:**

In the past two decades, healthcare adopted industrial strategies for process measurement and control. In the industry model, care is taken to avoid minimal deviations from a standard. In healthcare there is scarce data to support that a similar strategy can lead to better outcomes. Briefly, when compliance is high, further attempts to improve uptake of a process are seldom made. Our intensive care unit (ICU) improved the compliance with minimizing sedation from a high baseline of 80.4% (95% CI: 66.9 to 90.2) to 96.2% (95% CI: 95.2 to 97.0) 12 months after a quality improvement initiative. We sought to measure whether this minute improvement in compliance led to a reduction in duration of mechanical ventilation.

**Methods:**

We collected data on compliance with the process during 12 months. A trained data collector abstracted data from charts every other day. Our database contains data for length of mechanical ventilation, mortality, type of admission, and acute physiology and chronic health evaluation (APACHE) II scores for the 12 months before and after the process improvement.

To control for secular trends we used an interrupted-time series with adjustment for auto-correlation. We calculated the expected length of mechanical ventilation on each month by the end of the intervention period, and calculated the fitted value for the post-intervention months.

**Results:**

We included 1556 patients. There was an immediate effect of the intervention (regression coefficient = -0.129, P value < 0.001) and the secular trend was a determinant of length of mechanical ventilation (regression coefficient = 0.010, P value = 0.004). The trend post-intervention was not significant (regression coefficient = 0.004, P value = 0.380).

The relative change in the length of mechanical ventilation was 14.5% (IQR 13.8% to 15.8%) and the total expected decrease in mechanical ventilation days was 502.7 days (95% CI 300.9 to 729.1) over one year.

**Conclusions:**

In a system already working at high levels of compliance, outcomes can still be improved. Our intervention was successful in reducing the length of mechanical ventilation. ICUs should have a process of quality assurance in place to provide constant monitoring of key quality of care processes and correct deviations from the proposed standard.

## Introduction

Medical knowledge increases rapidly, making it increasingly difficult for clinicians to update their practice to allow the incorporation of new advances in care. Arguably the largest deficiency in modern health care is the frequent failure to adhere to evidence-based best practices [1]. These are practices related to the prevention, diagnosis and treatment of disease that have been demonstrated to improve clinically relevant outcomes. Both the government [2] and the healthcare industry allocate the majority of their resources towards the discovery of new interventions. In the United States the annual budget of the Agency for Healthcare Research and Quality, the federal agency responsible for research devoted to improve fidelity, is 1% of the budget for the National Institutes of Health [3], where the majority of money is allocated to research towards the discovery of new and more effective interventions. The assumption that new interventions are effectively adopted by healthcare systems may partially explain this disparity. However, studies have shown that as many as 30 to 50% of patients do not receive recommended evidence-based care and 20 to 30% of patients receive unnecessary interventions [1,4].

The problem is partly related to the lack of generalizable knowledge on how to effectively perform knowledge translation. Recent trials and meta-analysis demonstrate only modest, or even a lack of improvement, in evidence-based practices through the use of multifaceted programs to improve quality, including educational efforts, "bundle" tools, and audit and feedback [5-9]. Filling this gap in the implementation of available evidence, thus increasing system fidelity, can be a better opportunity to improve care than investigating for more effective interventions [10]. One of the approaches adopted in the industry to increase fidelity is the ongoing monitoring of processes of care, coupled with actions to improve quality even when minimal deviations from a standard are observed. This is usually performed with the aid of statistical process control charts, where deviations from a standard are differentiated from variation due to chance [11]. These charts are constructed with continued audits of "products" (in healthcare the "product" can be viewed as compliance with a process of care or the measurement of unwanted outcomes, such as nosocomial infections) and plotting of results over time. Depending on the characteristics of the variable (continuous or categorical) charts are constructed showing the variation over time and the standard error (SE) of the point estimates are used to create control lines at ± 2 SE and ± 3 SE, equivalent to 95% and 99% confidence intervals (CIs). Several rules are used to detect variation that is not due to chance, the most common ones being 1 point above or below the 3 SE control or 2 out of 3 points above or below the 2 SE control [12]. While large deviations from practice can trigger interventions in healthcare, there are scarce data to support a similar approach when the deviation is minimal, such as when there is reasonably high compliance with a process of care.

Strategies to limit the amount of sedation in critically ill patients, either by performing a daily interruption [13], or by minimizing the use of continuous infusions [14] reduce time on mechanical ventilation without causing harm. In one trial, benefits of duration of mechanical ventilation were clinically relevant, with a mean reduction of 2.4 days [13] and up to 4 extra ventilator-free days in a subsequent trial [14]. It is one of the most compelling examples of evidence-based medicine in critical care, and guidelines endorse the use of minimal sedation [15]. Ideally, every patient undergoing mechanical ventilation should have sedation minimized, as long as sedatives are not being used as treatment for neurological disorders. We have previously identified that in our unit, compliance with this evidence-based process was 80.4% (95% CI: 66.9 to 90.2). We undertook a quality improvement project with a goal of increasing compliance to 95% and we sought to investigate whether this small increment in compliance would result in further decreases in the duration of mechanical ventilation.

Given the limited efficacy of most interventions to improve quality of care [7] we sought first to understand barriers to current practice and used an interprofessional team to customize solutions to the barriers identified locally. The interprofessional group focused on theoretical approaches that have been demonstrated to increase compliance with evidence-based interventions:

(1) Reminders: Even when clinicians are aware of recommendations, they may forget to provide evidence-based interventions. A good example of this is the use of prophylaxis for deep venous thrombosis (DVT). This is an intervention where there is robust evidence and no disagreement. It is simple, not time consuming, and clinicians are aware of its importance and willing to use it. However, they may still forget to prescribe it. A randomized controlled trial of electronic reminders demonstrated a higher than two-fold increase in the use of DVT prophylaxis in hospitalized patients [16]. Reminders may also help with decreasing inertia, which is defined as lack of adoption of a process even in the face of knowledge and acceptance [17]. The presence of a reminder may increase compliance by reducing inertia in up to 40% of encounters [18].

(2) Simplification: simpler interventions have a lower threshold for adoption. Simpler interventions allow clinicians to easily understand their meaning and the rationale behind them. Also, clinicians perceive simple interventions to reduce their workload, which is an important barrier for implementation [19]. Almost 40% of physicians classified specific guidelines as "inconvenient" or difficult to use [20]. A systematic review identified guideline complexity, defined as when the average clinician perceives the guideline to be difficult to understand or to acquire the necessary skills, as responsible for 30% of the variation in compliance [21].

(3) Academic detailing: 'one-on-one' teaching by clinician champions seems to be particularly helpful. Two large systematic reviews support academic detailing as one of the most effective tools for behavioral change [22,23]. Academic detailing at the point of care may be particularly useful, as specific questions are answered and an immediate action is taken when the champion is available to help provide care.

## Materials and methods

### Setting

Sunnybrook Health Sciences Centre is a tertiary teaching hospital, affiliated with the University of Toronto. It has six critical care units including three level-3 units (where patients can be ventilated), namely a trauma-medical-surgical level-3 unit, a burn unit and a cardiovascular unit, and three level-2 units (where patients do not undergo invasive mechanical ventilation), namely a neuro-trauma unit, a medical-surgical unit and a coronary unit. Data for this study are based on admissions to the level-3 trauma-medical-surgical unit, which has 20 beds, the majority of patients cared for with a nurse ratio of 1:1. The nursing care delivery model is total patient care, which includes basic and advanced critical care core competencies. No other changes in the organization, relevant processes of care, or structure of the ICU took place at the same time.

### Quality Improvement Process

In April 2009 a protocol for sedation interruption was introduced in the level-3 units. In February 2010 we conducted an audit of compliance with the process, which revealed that 80.4% (95% CI: 66.9 to 90.2) of patients received an interruption of sedation or were given only boluses of sedatives as opposed to continuous infusions. An interprofessional team, consisting of physicians, nurses, respiratory therapists and pharmacists, was assembled to improve this process. The first step was to identify barriers and solutions from the end-users. We interviewed bedside nurses and respiratory therapists and identified 4 main barriers: (1) lack of knowledge of the protocol; (2) complexity of the protocol; (3) time to start sedation interruption considered unsafe and not realistic; (4) lack of accountability.

Based on the suggestions given by end-users, we designed a multi-faceted strategy to improve compliance with minimal sedation:

(1) Protocol design: the interprofessional team re-designed and simplified the protocol, outlining exclusion criteria and parameters for clinical assessment; the suggested time to interrupt sedation was also changed. We also clarified the misconception that a sedation interruption was only needed when patients were ready for extubation. Protocol redesign was a key step in addressing barriers 2 to 4 as the new protocol is simpler, more in line with accepted nursing practice, and establishes the bedside nurse as the clinician responsible for interrupting sedation in the morning.

(2) Reminder: we included a sedation interruption order in the admission pre-printed order set. In the first month we also used sticky-notes in the patients' charts to remind the team of patients who were receiving a continuous infusion of sedation. Reminders are important to address lack of knowledge, as they steer the clinician's attention to a required practice and also overcome inertia.

(3) Education: staff received in-service training, electronic material by e-mail and available on the intranet, training during staff meetings and at the point of care. During the first month of implementation, the critical care fellow, the respiratory therapist and the nurse in charge of the unit identified all eligible patients during the night shift. The next morning, the nurse educator, advanced practice nurse and/or nurse manager discussed the protocol directly with the bedside nurse. Questions were answered and if the nurse felt uncomfortable proceeding with the sedation interruption, the educator, advanced practice nurse or nurse manager encouraged the nurse and stayed at the bedside for troubleshooting. This interprofessional approach to identifying patients helped ensure that sedation was discussed at night and that patients were 'cleared' by physicians and respiratory therapists before the interruption. Education and especially the academic detailing provided at the point of care were instrumental in improving knowledge and accountability. The discussion of inclusion and exclusion criteria and troubleshooting the protocol at the bedside facilitates learning by using real situations.

### Ethics

As this project was undertaken to improve the quality of an already existing evidence-based process and benchmarking databases, the Research Ethics Board at Sunnybrook Health Sciences Centre waived the need for its review.

### Data Collection

The primary outcome was the length of mechanical ventilation. Data on length of mechanical ventilation were retrieved from a locally held quality benchmark database that has been in place since 2009 and has not undergone changes in data entry for this variable since its inception. We collected data from May 2009 through April 2011. The intervention took place in May 2010, yielding data for analysis from 12 months before and 12 months after the intervention. We included data only for the first ICU admission of each patient. Data for duration of mechanical ventilation were collected at the individual patient level.

Compliance with the evidence-based process: a trained data collector collected data on all ventilated patients every other day during weekdays, on the use of no sedation, boluses of sedation and continuous infusions of sedatives. Inclusion and exclusion criteria for sedation interruption were based on the protocol, as used in two previous randomized controlled trials [13,24]. If the patient was receiving a continuous infusion of sedatives and had no contraindications we recorded whether or not his sedation was interrupted. To calculate the compliance with the process we included in the denominator all patients who were ventilated and had no contraindications to have minimal sedation, and in the numerator all those who satisfied our definition of minimal sedation. We define minimal sedation as the use of boluses of sedatives without a continuous infusion or as an interruption in the continuous infusion of sedatives. Analgesics are not considered for sedation interruption and are titrated according to pain scales.

Data on severity of illness and type of admission was retrieved from an acute physiology and chronic health evaluation (APACHE II) database, in place for benchmarking before the quality improvement project was initiated.

### Statistical Analysis

**Figure 1:**
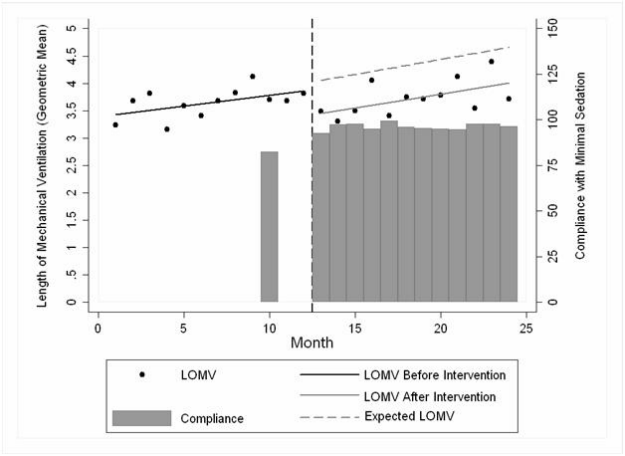
**Length of mechanical ventilation over time**. LOMV, Length of Mechanical Ventilation.

Data are presented as mean and standard deviation, median and interquartile range (IQR) or proportions, with a 95% CI. Our outcome of interest, time of mechanical ventilation, was log transformed to normalize the error terms, as it was positively skewed [25]. For ease of interpretation Figure 1 shows the exponentiated results, which represent the geometric mean.

Before- and after-studies may be biased by secular trends, and this bias may either show differences that are not real, or obscure real differences that exist. To control for secular trends we used an interrupted-time series with adjustment for autocorrelation using an autoregressive integrated moving average (ARIMA) model as described elsewhere [26]. Individual, log-transformed, patient data were aggregated by month to allow for ARIMA modeling. All regression models used maximum likelihood estimates and we controlled for autocorrelation by correcting for autoregressive effects. The interrupted time series is the strongest, quasi-experimental design to evaluate effects of time-delimited interventions [27]. Briefly, this involves a multilinear regression with two variables for time (β1 = time since inception of the cohort and β2 = time since the intervention, being 0 for all time-points before the intervention) and a variable for group (β3 = 0 before the intervention and 1 after the intervention). Interpretation of the regression involves looking at both the immediate change in level (β3) and the change in trend over time (β2). We checked for autocorrelation by visual inspection of autocorrelograms and partial autocorrelograms of the series and its residuals, and with the Box-Pierce Q statistic [28]. Time-series analysis requires a stationary process, which implies that the mean and variance do not change over time. When this assumption is not met, statistically significant results can be generated when no real signal exists. ARIMA models can use a differentiating function to create a stationary process. We checked the process was stationary using both the Phillips-Perron and the Kwiatkowski-Phillips-Schmidt-Shin (KPSS) tests [29,30].

We calculated the expected length of mechanical ventilation in each month by the end of the intervention period with data from the intercept, and β1 (time since cohort inception), and calculated the fitted value for the post-intervention months with the intercept, β1, β2 (time after the intervention), and β3 (level after intervention). The relative difference in these two points represents the effect of the intervention in each month. We have multiplied this difference by the number of patients in each month post-intervention to provide an estimate on the total amount of ventilator-days gained with the intervention. We assessed the fit of the model by examining residuals around the predicted lines. Normally distributed residuals that follow no observable pattern over time indicate that assumptions of the linear model are met [26].

All analysis was performed using STATA/SE 10.0 for Windows (StataCorp LP, TX, USA).

## Results

In this 2-year time period 1,826 patients were admitted to the level-3 medical-surgical unit. We excluded 108 patients who were never ventilated and 162 re-admissions, for a total of 1,556 patients included in the study (753 pre-intervention, 803 post-intervention). The median number of ventilated patients per month was 65 (IQR = 61 to 69). Baseline characteristics of the two groups are shown in Table 1. After the intervention there were more patients admitted under the trauma diagnostic category and fewer under the respiratory category. Otherwise, the two cohorts were similar in age, source of admission, APACHE II scores and mortality.

**Table 1:** Baseline characteristics.

Variable	Pre-Intervention (*n *= 753)	Post-Intervention (*n *= 803)	*P*-value
Age, years (mean ± SD)	59.4 ± 20.8	58.2 ± 20.3	0.26
Sex, male (%)	63.5	61.9	0.54
Source			0.11
Emergency (%)	43.4	43.3	
Ward (%)	30.6	29.5	
Other (%)	26.0	27.2	
Diagnostic category			0.04
Trauma (%)	29.6	35.3	
Respiratory (%)	27.7	22.6	
Neuro (%)	11.7	13.8	
Gastrointestinal (%)	7.8	6.1	
Cardiovascular (%)	6.9	6.0	
Other (%)	16.3	16.2	
Length of mechanical ventilation, days (mean ± SD)	6.5 ± 10.9	6.1 ± 8.1	NA
Ventilator-free days (mean ± SD)	16.9 ± 10.9	17.2 ± 10.7	NA
Apache II score (mean ± SD)	22.3 ± 7.9	22.3 ± 8.6	0.97
Mortality, % (95% CI)	23.5 (20.5, 26.7)	22.2 (19.3, 25.2)	0.54

APACHE, acute physiology and chronic health evaluation; NA, not applicable

### Compliance with the quality improvement intervention

During the 12 months after the intervention we observed that patients received minimal sedation in 1,921 patient-days, from a total of 1,997 eligible patient-days (96.2%, 95% CI 95.2 to 97.0).

### ARIMA Modeling

The Phillips-Perron and KPSS tests demonstrated stationarity of the series (Phillips-Perron, *P *= 0.0000, rejecting the null hypothesis on non-stationarity; KPSS, *P *> 0.1, confirming the null hypothesis of stationarity), therefore no differentiating function was introduced in the ARIMA model. Neither the visual inspection of correlograms nor the Box-Pierce Q statistic suggested autocorrelation. An ARIMA model with zero lags of autocorrelation, however, demonstrated one lag of autocorrelation in the residuals. Therefore we modeled our data with one lag of autocorrelation. The point estimates of the main effects did not change between models with or without autocorrelation adjustment, but the residuals of the model with one lag of autocorrelation showed no more autocorrelation.

### Length of Mechanical Ventilation

There was an immediate effect of the intervention (regression coefficient = -0.129, *P *< 0.001) and the secular trend (β1) was a determinant of length of mechanical ventilation (regression coefficient = 0.010, *P *= 0.004). The trend post-intervention (β2) was not significant (regression coefficient = 0.004, *P *= 0.380) (Figure 1).

The median relative change in the fitted length of mechanical ventilation was 14.5% (IQR 13.8 to15.8) and the total expected decrease in mechanical ventilation days was 502.7 days (95% CI 300.9 to 729.1) over one year.

Due to the differences observed in the diagnostic categories between the two time periods we reanalyzed the data restricting the observations to respiratory/non-respiratory admission and to trauma/non-trauma admissions (Table 2). In the model excluding respiratory admissions we could not find any signal of a secular trend or changes in trend and level after the intervention (full model, *P *= 0.2), while the analysis limited to respiratory admissions demonstrated an immediate effect of the intervention (regression coefficient = -0.383, *P *< 0.03). The model excluding trauma admissions also demonstrated an immediate effect of the intervention (regression coefficient = -0.215, *P *< 0.001), a secular trend towards an increase in length of mechanical ventilation (regression coefficient = 0.011, *P *< 0.001), and a post-intervention trend towards an increase in length of mechanical ventilation (regression coefficient = 0.013, *P *< 0.032), while the model limited to trauma admissions demonstrated no signal (full model, *P *= 0.29).

**Table 2:** Changes in length of mechanical ventilation.

	Full cohort	Cohort excluding respiratory admissions	Cohort of respiratory admissions	Cohort excluding trauma admissions	Cohort of trauma admissions
Secular trend (β1)	0.010^a^	0.001	0.025	0.011^b^	0.009
Trend post-intervention (β2)	0.004	0.001	0.020	0.013^a^	-0.009
Immediate effect (β3)	-0.129^b^	0.004	-0.383^a^	-0.215^b^	0.009

^a^*P *< 0.05; ^b^*P *< 0.001

## Discussion

Our results support the conclusion that a quality improvement intervention can reduce the length of mechanical ventilation even when baseline compliance is already high. Our cohort demonstrated a trend towards an increased length of mechanical ventilation over time (reflected by a positive coefficient on β1). We can only speculate on the possible reasons for this increase: if clinicians are changing their practice towards being more conservative in making decisions to withhold or withdraw life support [31], such as after neurological trauma or cardiac arrest, or in patients with cancer [32], we can expect to see an increase in time on ventilation. Another possibility is a change in severity of illness over time, which we could not demonstrate from our limited data with APACHE scores. Interestingly, two reports of time trends in mechanical ventilation suggest a similar increase in duration of mechanical ventilation over past years [33,34].

We were able to demonstrate the effectiveness of our intervention: there was a change in level (negative coefficient for β3), implying an immediate effect of the intervention, which did not reverse after the intervention (reflected by a non-significant coefficient on β2). We interpret these results as an indication that there was a persistent effect of the intervention over time. If, as is the case with so many quality improvement interventions, these effects were transient, we would expect to see a positive and statistically significant coefficient on analysis of trends post-intervention (β2). Our study may have been underpowered to detect this return to baseline, but we can conclude that at least over a period of 12 months, the intervention was effective in reducing the length of mechanical ventilation.

One of the limitations in our study is the difference in admission categories between the two time periods. While we could not formally adjust the ARIMA models for the types of admissions, we report results for the same modeling for the main diagnostic categories of respiratory illness and trauma. The cohort of patients with respiratory problems shows the same immediate effect of our intervention, without a return to baseline, and there was no identifiable effect in the trauma cohort. While we can offer no data to address the reasons for these differences, one may consider that patients admitted due to respiratory causes were more likely to be over-sedated and benefited from the intervention, while trauma patients may have two distinctive patterns of sedation use: the need for sedation to control intracranial pressure or the use of minimal amounts of sedatives for a short period of mechanical ventilation. In both cases it is possible that a protocol for sedation interruption would have no effect, as patients with intracranial hypertension do not undergo sedation interruption and patients who undergo a short period of mechanical ventilation may already be using minimal doses of sedatives. Importantly, given that an effect is seen in the respiratory group, a lower number of admissions of this group in the second part of the study should bias our results towards the null hypothesis, as there would be fewer patients in whom the intervention is effective. This imbalance therefore, strengthens our results for the whole cohort. We would caution against the interpretation that the intervention was only effective in respiratory cases, as the statistical model may not perform as well with the smaller numbers of patients in the subgroups that were analyzed.

An important limitation of our findings relates to the environment where this study took place. In Ontario, most critical care units capable of caring for ventilated patients are staffed to provide the majority of patients with one-to-one nursing care, which may not be the reality in other healthcare settings [35]. However, nurses in Ontario have responsibilities, such as bathing, mobilizing, suctioning and dialyzing patients, that may be performed by different clinicians or non-clinicians in other ICUs. Given this perspective, it is difficult to understand whether units with less intensive staffing will be able to safely reach high compliance with minimal sedation. Also, our unit has 20 beds with a high occupancy (> 100%) and mainly cares for acute care, ventilated patients (those transferred to a step-down unit when they no longer require invasive mechanical ventilation). Therefore, the exposure to the new protocol was facilitated by a process that occurs frequently (on average 3 to 4 patients were using continuous infusions of sedation every morning in the initial implementation month); in smaller and less acute units it may be difficult to have such intense exposure to a new process and the implementation period can be much longer. One of the main limitations of this paper is its use of a customized strategy to improve compliance. Our approach focused on understanding the barriers at the local level and applying methodologies that were tailored directly to address the local barriers. This is clearly not generalizable, but yet, may also be one of the strongest components of this paper: quality improvement projects may need to be customized to organizations. Consider that two different patients with similar symptoms (dyspnea) may have different diseases (congestive heart failure and asthma, for example), requiring different treatments; a trial that investigates a drug to treat dyspnea is unlikely to show benefit. Similarly, two organizations with the same problem (lack of compliance with minimal sedation) may have different causes (insufficient human resources or a strong predisposition against minimizing sedation) that may require different approaches [36].

Although we would not consider our results generalizable, the components of our approach may be transportable to other environments: reminders are a simple and effective tool in several different settings, ranging from DVT prophylaxis [16] to checklist prompts during rounds [37]; simplification of processes is used in the industry to improve efficiency and reduce errors; and academic detailing is suggested to be one of the few relevant knowledge translation methods in the current literature [22,23]. Although the use of these processes was customized to our unit (how to simplify the process, how to create the reminders and the daily process of academic detailing), their basic characteristics can be easily customized to most critical care units.

Our study also highlights another important aspect of research in quality improvement. Had we taken an approach to simply measure length of mechanical ventilation before and after we might have concluded that there was no effect of the intervention. Because the trend was towards an increased length of mechanical ventilation, any benefits would have been upset by this trend. Interrupted-time series analysis has been recommended to avoid identifying associations that are not real and only due to secular trends, but it can also be helpful to identify effective interventions when the trend is in the opposite direction. Interrupted time-series is a stronger research design than before- and after-studies to assess the effect of quality improvement interventions. However they are frequently poorly reported or analyzed in the literature. A review of 58 studies using interrupted time-series demonstrated that two-thirds were unable to rule out significant threats to internal validity. Thirty-three of these studies could be reanalyzed, and 8 were found to have no real effect [38]. Our study meets all quality criteria as described in this review: (1) intervention occurred independently of other changes over time; (2) sources of data collection were the same in both periods; (3) the primary outcome is objective and reliable (duration of mechanical ventilation); (4) all patients are included in the study; (5) there is good evidence that the intervention is linked to the outcome; (6) there are enough data points to detect a significant decay after the intervention and (7) we tested and adjusted for autocorrelation.

Although our unit was already compliant in more than 80% of patients, our small increase in compliance, just slightly above 15%, led to important gains in efficiency for the healthcare system: considering only our unit, we decreased the amount of mechanical ventilation by the equivalent of more than 500 days. Considering that most ICU costs are fixed (staffing, equipment, infra-structure costs) [39], this ICU would be able to care for an extra 125 patients (assuming an average 4 days of mechanical ventilation), with minimal changes in cost, after the intervention.

## Conclusions

A quality improvement intervention to increase compliance with minimal sedation is effective in reducing the length of mechanical ventilation. Monitoring and redesigning processes of care can increase compliance above 95%. Gains for the healthcare system were achieved in a unit that was already compliant with this evidence-based process. Further research should concentrate in identifying the most effective strategies to quickly implement and monitor evidence-based processes.

## Key messages

• A multifaceted strategy improved compliance with minimization of sedation

• Small improvements in compliance with minimizing sedation led to reduced duration of mechanical ventilation

• Quality assurance monitoring is an important component of quality improvement and ICUs should monitor its' key quality processes on a continued basis

## Abbreviations

APACHE: acute physiology and chronic health evaluation; ARIMA: autoregressive integrated moving average; DVT: deep venous thrombosis; ICU: intensive care unit; KPSS tests: Kwiatkowski-Phillips-Schmidt-Shin tests; LOMV: Length of Mechanical Ventilation; SE: standard error.

## Competing interests

The authors have no financial or non-financial competing interests related to the content of this article.

## Authors' contributions

ACKBA was involved in the design of the database to measure compliance with processes of care, survey of barriers to adoption of minimal sedation, redesign of the protocol for minimizing sedation, linking and analyzing the databases, and writing the manuscript. LK was involved with the survey of barriers to adoption of minimal sedation, redesign of the protocol for minimizing sedation, and writing the manuscript. AJ was involved with the survey of barriers to adoption of minimal sedation, redesign of the protocol for minimizing sedation, and writing the manuscript. All authors read and approved the final manuscript

## References

[B1] Chassin MR Galvin RW The urgent need to improve health care quality. Institute of Medicine National Roundtable on Health Care Quality JAMA 1998 280 1000 1005 10.1001/jama.280.11.1000 9749483

[B2] Sung NS Crowley WF Genel M Salber P Sandy L Sherwood LM Johnson SB Catanese V Tilson H Getz K Larson EL Scheinberg D Reece EA Slavkin H Dobs A Grebb J Martinez RA Korn A Rimoin D Central Challenges Facing the National Clinical Research Enterprise JAMA 2003 289 1278 1287 10.1001/jama.289.10.1278 12633190

[B3] Clancy CM AHRQ's FY 2005 Budget Request: New Mission, New Vision Health Serv Resh 2004 39 11 18

[B4] Schuster MA McGlynn EA Brook RH How good is the quality of health care in the United States? Milbank Q 2005 83 843 895 10.1111/j.1468-0009.2005.00403.x 16279970 PMC2690270

[B5] Hsu DJ Stone RA Obrosky DS Yealy DM Meehan TP Fine JM Graff LG Fine MJ Predictors of timely antibiotic administration for patients hospitalized with community-acquired pneumonia from the cluster-randomized EDCAP trial Am J Med Sci 2010 339 307 313 20224313 10.1097/MAJ.0b013e3181d3cd63 PMC2875077

[B6] Ferrer R Artigas A Levy MM Blanco J Gonzalez-Diaz G Garnacho-Montero J Ibanez J Palencia E Quintana M de la Torre-Prados MV for the Edusepsis Study G Improvement in Process of Care and Outcome After a Multicenter Severe Sepsis Educational Program in Spain JAMA 2008 299 2294 2303 10.1001/jama.299.19.2294 18492971

[B7] Grimshaw JM Thomas RE MacLennan G Fraser C Ramsay CR Vale L Whitty P Eccles MP Matowe L Shirran L Wensing M Dijkstra R Donaldson C Effectiveness and efficiency of guideline dissemination and implementation strategies Health Technol Assess 2004 8 3 72 10.3310/hta8060 14960256

[B8] Saint S Hofer TP Rose JS Kaufman SR McMahon LF Jr Use of critical pathways to improve efficiency: a cautionary tale Am J Manag Care 2003 9 758 765 14626473

[B9] Walsh JM McDonald KM Shojania KG Sundaram V Nayak S Lewis R Owens DK Goldstein MK Quality improvement strategies for hypertension management: a systematic review Med Care 2006 44 646 657 10.1097/01.mlr.0000220260.30768.32 16799359

[B10] Woolf SH Johnson RE The break-even point: when medical advances are less important than improving the fidelity with which they are delivered Ann Fam Med 2005 3 545 552 10.1370/afm.406 16338919 PMC1466946

[B11] Benneyan JC Lloyd RC Plsek PE Statistical process control as a tool for research and healthcare improvement Qual saf health care 2003 12 458 464 10.1136/qhc.12.6.458 14645763 PMC1758030

[B12] Finison LJ Finison KS Bliersbach CM The use of control charts to improve healthcare quality J Healthcare qual 1993 15 9 23 10.1111/j.1945-1474.1993.tb00073.x 10123345

[B13] Kress JP Pohlman AS O'Connor MF Hall JB Daily interruption of sedative infusions in critically ill patients undergoing mechanical ventilation The New Engl J Med 2000 342 1471 1477 10.1056/NEJM200005183422002 10816184

[B14] Strom T Martinussen T Toft P A protocol of no sedation for critically ill patients receiving mechanical ventilation: a randomised trial Lancet 2010 375 475 480 10.1016/S0140-6736(09)62072-9 20116842

[B15] Dellinger RP Levy MM Carlet JM Bion J Parker MM Jaeschke R Reinhart K Angus DC Brun-Buisson C Beale R Calandra T Dhainaut JF Gerlach H Harvey M Marini JJ Marshall J Ranieri M Ramsay G Sevransky J Thompson BT Townsend S Vender JS Zimmerman JL Vincent JL Surviving Sepsis Campaign: international guidelines for management of severe sepsis and septic shock: 2008 Crit Care Med 2008 36 296 327 10.1097/01.CCM.0000298158.12101.41 18158437

[B16] Kucher N Koo S Quiroz R Cooper JM Paterno MD Soukonnikov B Goldhaber SZ Electronic alerts to prevent venous thromboembolism among hospitalized patients New Engl J Med 2005 352 969 977 10.1056/NEJMoa041533 15758007

[B17] Main DS Cohen SJ DiClemente CC Measuring physician readiness to change cancer screening: preliminary results Am J Prev Med 1995 11 54 58 7748587

[B18] Lazaro P Murga N Aguilar D Hernandez-Presa MA Therapeutic inertia in the outpatient management of dyslipidemia in patients with ischemic heart disease. The inertia study Rev Esp Cardiol 2010 63 1428 1437 21144403 10.1016/s1885-5857(10)70277-2

[B19] Toma A Bensimon CM Dainty KN Rubenfeld GD Morrison LJ Brooks SC Perceived barriers to therapeutic hypothermia for patients resuscitated from cardiac arrest: a qualitative study of emergency department and critical care workers Crit Care Med 2010 38 504 509 10.1097/CCM.0b013e3181cb0a02 20016377

[B20] Cabana MD Rand CS Powe NR Wu AW Wilson MH Abboud PA Rubin HR Why Don't Physicians Follow Clinical Practice Guidelines? A Framework for Improvement JAMA 1999 282 1458 1465 10.1001/jama.282.15.1458 10535437

[B21] Grilli R Lomas J Evaluating the message: the relationship between compliance rate and the subject of a practice guideline Med Care 1994 32 202 213 10.1097/00005650-199403000-00002 8145598

[B22] Davis DA Thomson MA Oxman AD Haynes RB Changing physician performance. A systematic review of the effect of continuing medical education strategies JAMA 1995 274 700 705 10.1001/jama.1995.03530090032018 7650822

[B23] Oxman AD Thomson MA Davis DA Haynes RB No magic bullets: a systematic review of 102 trials of interventions to improve professional practice CMAJ 1995 153 1423 1431 7585368 PMC1487455

[B24] Girard TD Kress JP Fuchs BD Thomason JW Schweickert WD Pun BT Taichman DB Dunn JG Pohlman AS Kinniry PA Jackson JC Canonico AE Light RW Shintani AK Thompson JL Gordon SM Hall JB Dittus RS Bernard GR Ely EW Efficacy and safety of a paired sedation and ventilator weaning protocol for mechanically ventilated patients in intensive care (Awakening and Breathing Controlled trial): a randomised controlled trial Lancet 2008 371 126 134 10.1016/S0140-6736(08)60105-1 18191684

[B25] Hoaglin DC Mosteller F Tukey JW Understanding robust and exploratory data analysis 1983 New York: John Wiley & Sons, Inc

[B26] Wagner AK Soumerai SB Zhang F Ross-Degnan D Segmented regression analysis of interrupted time series studies in medication use research J Clin PharmTher 2002 27 299 309 10.1046/j.1365-2710.2002.00430.x 12174032

[B27] Gillings D Makuc D Siegel E Analysis of interrupted time series mortality trends: an example to evaluate regionalized perinatal care Am J Public Health 1981 71 38 46 10.2105/AJPH.71.1.38 7258429 PMC1619708

[B28] Box GEP Pierce DA Distribution of residual autocorrelations in autoregressive-integrated moving average time series models J Am Stat Assoc 1970 65 1509 1526

[B29] Kwiatkowski D Phillips PCB Schmidt PJ Shin Y Testing the null hypothesis of stationarity against the alternative of a unit root: how sure are we that economic time series have a unit root J Econom 1992 54 159 178 10.1016/0304-4076(92)90104-Y

[B30] Phillips PCB Perron P Testing for a unit root in time series regression Biometrika 1988 75 335 346 10.1093/biomet/75.2.335

[B31] Bertolini G Boffelli S Malacarne P Peta M Marchesi M Barbisan C Tomelleri S Spada S Satolli R Gridelli B Lizzola I Mazzon D End-of-life decision-making and quality of ICU performance: an observational study in 84 Italian units Intensive Care Med 2010 36 1495 1504 10.1007/s00134-010-1910-9 20464541

[B32] Lecuyer L Chevret S Thiery G Darmon M Schlemmer B Azoulay E The ICU trial: a new admission policy for cancer patients requiring mechanical ventilation Crit Care Med 2007 35 808 814 10.1097/01.CCM.0000256846.27192.7A 17235261

[B33] Esteban A Ferguson ND Meade MO Frutos-Vivar F Apezteguia C Brochard L Raymondos K Nin N Hurtado J Tomicic V Gonzalez M Elizalde J Nightingale P Abroug F Pelosi P Arabi Y Moreno R Jibaja M D'Empaire G Sandi F Matamis D Montanez AM Anzueto A for the VG Evolution of Mechanical Ventilation in Response to Clinical Research Am J Respir Crit Care Med 2008 177 170 177 17962636 10.1164/rccm.200706-893OC

[B34] Needham DM Bronskill SE Calinawan JR Sibbald WJ Pronovost PJ Laupacis A Projected incidence of mechanical ventilation in Ontario to 2026: Preparing for the aging baby boomers Crit Care Med 2005 33 574 579 10.1097/01.CCM.0000155992.21174.31 15753749

[B35] Penoyer DA Nurse staffing and patient outcomes in critical care: A concise review Crit Care Med 2010 38 1521 1529 10.1097/CCM.0b013e3181e47888 20473146

[B36] Amaral AC A window of opportunity for collaboration between intensivists and oncologists J Crit Care 2011 in press 10.1016/j.jcrc.2011.07.072 21958980

[B37] Weiss CH Moazed F McEvoy CA Singer BD Szleifer I Amaral LAN Kwasny M Watts CM Persell SD Baker DW Sznajder JI Wunderink RG Prompting physicians to address a daily checklist and process of care and clinical outcomes: a single-site study Am J Respir Crit Care Med 2011 184 680 686 10.1164/rccm.201101-0037OC 21616996 PMC3208596

[B38] Ramsay CR Matowe L Grilli R Grimshaw JM Thomas RE Interrupted time-series designs in health technology assesment: lessons from two systematic reviews of behavior change strategies Int J Technol Assess Health Care 2003 19 613 623 15095767 10.1017/s0266462303000576

[B39] Burchardi H Schneider H Economic aspects of severe sepsis: a review of intensive care unit costs, cost of illness and cost effectiveness of therapy Pharmacoeconomics 2004 22 793 813 10.2165/00019053-200422120-00003 15294012

